# THE PANDEMIC AS A GREAT UNEQUALIZER? RURAL–URBAN DIVIDE IN COVID-19 CONCERNS AND OLDER ADULTS’ MENTAL HEALTH IN CHINA

**DOI:** 10.1093/geroni/igae098.2342

**Published:** 2024-12-31

**Authors:** Jingwen Liu, Yang Han

**Affiliations:** Rice University, Houston, Texas, United States; The Chinese University of Hong Kong, Hong Kong, China (People’s Republic)

## Abstract

Hukou is a household registration system that divides individuals to rural or urban residents at birth based on their parents’ status, which significantly contribute to health disparities in China. This paper investigates whether older adults’ concerns about the Covid-19 pandemic exacerbate rural-urban disparities and the potential mechanism of coping behaviors. Using the 2020 wave of China Health and Retirement Longitudinal Study (CHARLS), we employed OLS and KHB analyses to local rural residents (rural-Hukou holders living in rural areas, N=9,101), rural-urban migrants (rural-Hukou holders living in urban areas; N=2,418), and local urban residents (urban-Hukou holders living in urban areas, N=2,874), respectively. Results suggest that local rural residents and rural-urban migrants both show substantially higher Covid concerns and depressive symptoms than local urban residents. Covid concerns are associated with higher depressive symptoms among all groups, but the association is statistically more prominent among local rural than local urban residents. While changes in health habits and in-person meetings contribute to explaining these associations for both groups, the delayed medical services and increased preventive behaviors exclusively explain the association for local rural residents. Rural-urban migrants exhibit a dual profile: they demonstrate higher concerns like rural residents while adopting fewer coping behaviors parallel to urban residents. This paper confirms that Hukou acts as a fundamental cause of rural-urban health disparities that will be reproduced and reinforced during societal crises like the Covid pandemic. Rural-born individuals’ health disadvantages may be reduced but not eliminated by the migration to urban areas without substantial reforms of Hukou system.

